# Methylation of microRNA-129-5P modulates nucleus pulposus cell autophagy by targeting Beclin-1 in intervertebral disc degeneration

**DOI:** 10.18632/oncotarget.21137

**Published:** 2017-09-21

**Authors:** Kangcheng Zhao, Yukun Zhang, Liang Kang, Yu Song, Kun Wang, Shuai Li, Xinghuo Wu, Wenbin Hua, Zengwu Shao, Shuhua Yang, Cao Yang

**Affiliations:** ^1^ Department of Orthopaedics, Union Hospital, Tongji Medical College, Huazhong University of Science and Technology, Wuhan 430022, China

**Keywords:** miR-129-5P, intervertebral disc degeneration, Beclin-1, autophagy, methylation

## Abstract

MicroRNAs play an important role in the etiology and progression of many diseases, including intervertebral disc degeneration (IVDD). The miRNA miR-129-5P regulates autophagy in various cancers, but its role in human nucleus pulposus (NP) cells is unclear. The present study investigated whether miR-129-5p regulates the expression of Beclin-1 which is known to induce autophagy in NP cells by evaluating their levels in normal and degenerative disc tissues and human NP cells transfected with miR-129-5P mimic or inhibitor by quantitative real-time (qRT-)PCR, western blotting, flow cytometry, and immunofluorescence analysis. A bioinformatics analysis was used to predict the relationship between miR-129-5P and Beclin-1, which was confirmed by the dual luciferase assay. DNA methylation status was assessed by methylation-specific PCR, and the effect of demethylation on miR-129-5P level and autophagy was examined by qRT-PCR, western blotting, and flow cytometry. We found that miR-129-5P expression was downregulated while that of Beclin-1 and LC3-II was upregulated in degenerative disc NP cells. Meanwhile, autophagy was reduced in human NP cells transfected with miR-129-5P mimic, whereas the opposite result was observed upon treatment with miR-129-5P inhibitor. Bioinformatics analysis and the luciferase reporter assay revealed that Beclin-1 is a target of and is inhibited by miR-129-5P. We also found that CpG islands in the miR-129-5P promoter region were hypermethylated in degenerative as compared to normal disc tissue. Thus, miR-129-5P blocks NP cell autophagy by directly inhibiting Beclin-1, a process that is dependent on miR-129-5P promoter methylation.

## INTRODUCTION

More than half of individuals experience lower back pain during their lifetime [1], which is frequently associated with intervertebral disc degeneration (IVDD). Although not lethal, IVDD is debilitating and constitutes a significant burden on society [2, 3]. IVDs are the soft tissue between vertebrae that absorb and distribute applied loads and lend flexibility to the spine [4, 5]. Spinal instability and structural changes caused by increased inflammatory cytokines and decreased hydrophilic matrix molecules are the main causes of herniation, sciatica, and stenosis [6]. The abnormal production of pro-inflammatory cytokines secreted by disc cells [7, 8] as a result of genetic predisposition, smoking, infection, excessive biomechanical loading, decreased nutrient transport, and aging [9–13] triggers pathogenic responses in disc cells including autophagy, senescence, and apoptosis [9, 14, 15] that contribute to IVD degeneration [16, 17].

The dysregulation of cell death mechanisms is implicated in the etiology and pathogenesis of diseases such as cancer, heart disease, Parkinson’s and Alzheimer’s diseases, and disc degeneration [18–20]. Autophagy is a conserved and ubiquitous form of cytoprotection that degrades unnecessary or dysfunctional cellular components to maintain homeostasis [20, 21] and protects against apoptosis [16]; it consists of initiation, elongation, maturation, and lysosomal fusion steps [17, 22] that are regulated by specific genes. For example, Beclin-1 (also known as autophagy-related Atg6) and microtubule-associated protein 1 light chain (LC)3 (also known as Atg8) are required for autophagosome formation [15]. Beclin-1 is a member of the B cell lymphoma (Bcl)-2 gene family that promotes autophagy in mammalian cells [23]. Beclin-1 dependent autophagy has been reported in human nucleus pulposus [16, 24]. LC3 exists in two forms, LC3-I in the cytoplasm and LC3-II that binds to the autophagosome membrane. LC3-I is converted to LC3-II during autophagy progression, which can be triggered by oxidative stress, hypoxia, nutrient deprivation, and mechanical compression. It was recently reported that autophagy was increased in rat nucleus pulposus (NP) cells of IVDD tissue [25, 26].

Apoptosis is a form of programmed cell death that is stimulated by inflammatory, injury, DNA damage, and oxidative stress [17, 27–29]. Apoptosis has been observed in IVDD [20, 30]; recent studies have shown that this can be inhibited by autophagy [20, 31]. Others have reported that decreasing endoplasmic reticulum stress by autophagy prevented apoptosis [32], although the underlying mechanism is unclear. We previously found that the fusion of autophasosomes and lysosomes is a key event in the process of autophagy, and that cathepsins in the lysosome regulate apoptosis [33, 34]. We therefore speculated that autophagy regulates these cathepsins and thereby prevents apoptosis in human degenerative NP cells.

Micro (mi)RNAs are endogenous noncoding RNA molecules with a length of about 22 nucleotides that post-transcriptionally regulate gene expression through base pairing with the 3′-untranslated region (UTR) of target mRNA [35]. MiRNAs are involved in the control of cell proliferation, cycling, apoptosis, and invasion [36–38], and their dysregulation is linked to many human diseases. A recent study showed that miR-129-5P modulates the expression of Beclin-1 [39] and regulates autophagy in atherosclerosis. However, the role of miR-129-5P in the progression of IVDD is unclear.

DNA methylation silences the expression of miRNAs and is considered as a potential therapeutic strategy for cancer treatment [40, 41]. For example, methylation-induced miR-1247 silencing promotes cancer cell invasion and migration in non-small cell lung cancer [42], while miR-129-5P methylation was associated with expression of human valosin-containing protein in osteosarcoma [43]. Based on these findings, we speculated that miR-129-5P modulates IVDD progression via DNA methylation. To test this hypothesis, the present study investigated the relationship between miR-129-5P methylation status and expression and autophagy in NP cells.

## RESULTS

### MiR-129-5P and Beclin-1 show opposite expression patterns in degenerative NP cells

**Figure 1:**
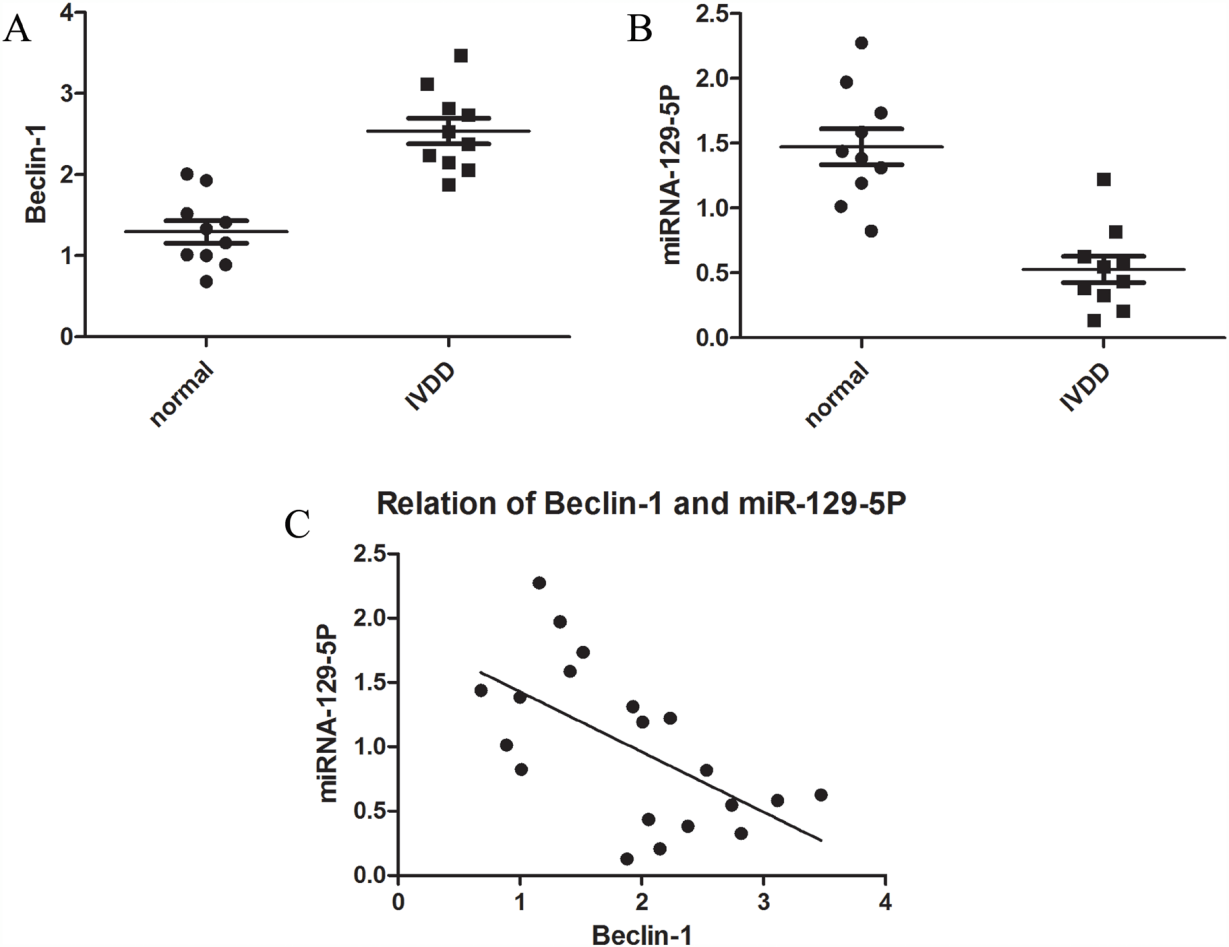
MiR-129-5P and Beclin-1 expression in normal and degenerative NP tissue **(A)** Beclin-1 mRNA level was upregulated in degenerative as compared to normal NP tissue. **(B)** MiR-129-5P level was downregulated in degenerative as compared to normal NP tissue, as determined by qRT-PCR. **(C)** Correlation between miR-129-5P and Beclin-1 mRNA levels in NP tissue.

To investigate the biological roles of miR-129-5P and Beclin-1 in IVDD, we evaluated miR-129-5P and Beclin-1 expression in normal and degenerative human NP cells by quantitative real-time (qRT-)PCR (Figure 1A, 1B). We found that miR-129-5P expression was downregulated whereas Beclin-1 transcript level was upregulated in degenerative as compared to normal disc tissue (Figure 1C). These results suggest that miR-129-5P and Beclin-1 are involved in the progression of disc degeneration.

### MiR-129-5P suppresses autophagy in degenerative NP cells

**Figure 2:**
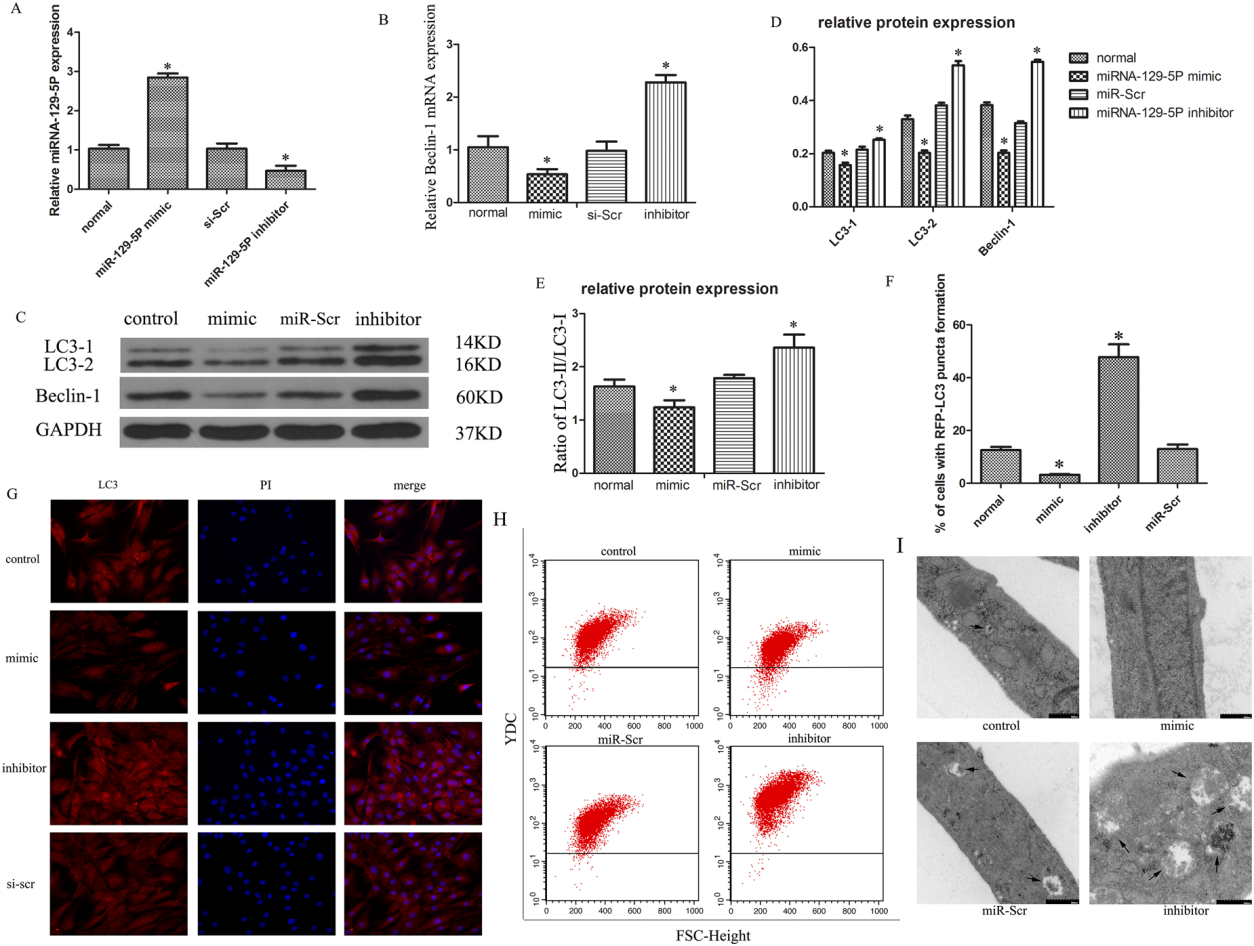
MiR-129-5P modulates autophagy in degenerative human NP cells Cells were transfected with miR-129-5P mimic, miR-129-5P inhibitor, or scrambled mi-129-5P (miR-Scr) for 48 h. **(A)** MiR-129-5P expression was evaluated by qRT-PCR. **(B)** LC3 and Beclin-1 mRNA, as determined by qRT-PCR. **(C, D)** LC3-II, LC3-I, and Beclin-1 protein expression in human NP cells, as determined by western blotting. **(E)** LC3-II and LC3-I ratio was decreased and increased upon treatment with mimic and inhibitor, respectively. **(F, G)** LC3 expression was detected by Immunofluorescence analysis in transfected human NP cells. **(H)** Autophagy in treated NP cells, as determined by flow cytometry. **(I)** Autophasosomes and autolysosmes in NP cells were determined by electron microscope. Results are shown as mean ± SD. Data are representative of three independent experiments. *P < 0.05 vs. miR-Scr.

Autophagic flux can be measured by the ratio of LC3-II to LC3-I [44]. NP cells were transfected with miR-129-5P mimic or inhibitor with high transfection efficiency being observed (Figure 2A). Beclin-1 mRNA levels were downregulated in cells transfected with miR-129-5P mimic as compared to the control (Figure 2B). The opposite was observed upon transfection of miR-129-5P inhibitor (Figure 2B). LC3-II and Beclin-1 protein expression was markedly decreased in the miR-129-5P mimic-transfected group as compared to the control and inhibitor-treated groups (Figure 2C, 2D). Thus, the ratio of LC3-II to LC3-I was decreased in cells transfected with miR-129-5P mimic relative to the control or inhibitor-transfected groups (Figure 2E). Moreover, LC3 expression was higher in the inhibitor-transfected group (Figure 2F, 2G), and a flow cytometry analysis revealed that autophagic activity was increased (Figure 2H). An electron microscopy analysis revealed more autophagosomes and autophalysosomes in cells transfected with miR-129-5P inhibitor as compared to the mimic (Figure 2I). These results indicate that miR-129-5P suppresses human NP cell autophagy.

### MiR-129-5P suppresses Beclin-1 expression in NP cells

**Figure 3:**
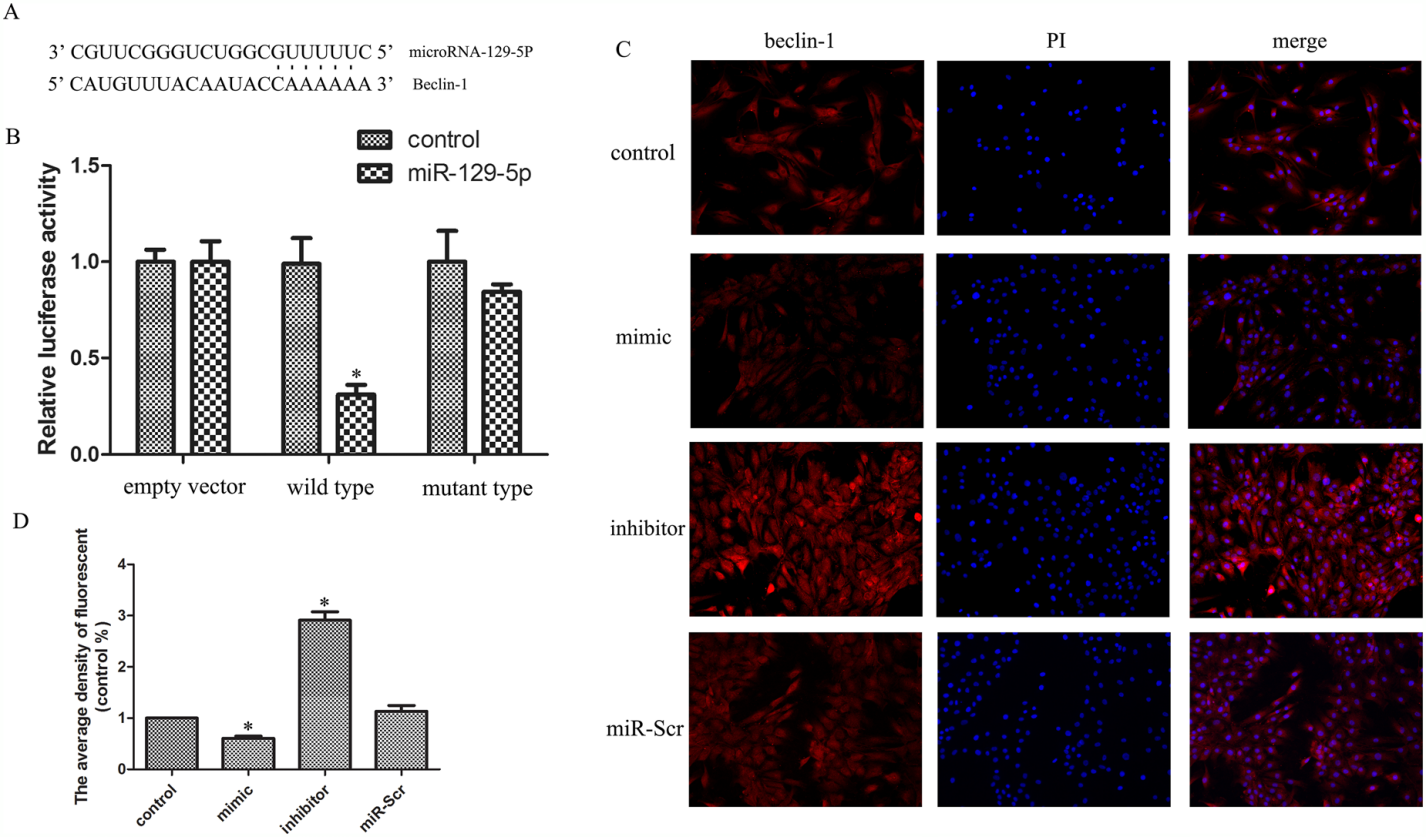
MiR-129-5P regulates Beclin-1 expression in degenerative human NP cells **(A)** Putative miR-129-5P target site in the 3′-UTR of Beclin-1 transcript predicted by bioinformatics analysis. **(B)** Luciferase activity in human NP cells co-transfected with miR-Scr or miR-129-5P, as measured by a luciferase reporter assay. **(C, D)** Immunofluorescence analysis of LC3-II expression in human NP cells transfected with miR-129-5P mimic, miR-129-5P, inhibitor, or scrambled mi-129-5P. Results are shown as mean ± SD. Data are representative of three independent experiments. *P < 0.05 vs. control.

A bioinformatics analysis revealed a miR-129-5P seed sequence in the 3′-UTR of Beclin-1, an autophagy-related gene (Figure 3A). We therefore speculated that miR-129-5P is involved in NP cell autophagy. The results of the dual-luciferase reporter assay showed that luciferase activity was decreased in miR-129-5P-transfected cells; this effect was abrogated when the binding site was deleted (Figure 3B). To clarify the role of miR-129-5P in NP cell autophagy, we inhibited or overexpressed miR-129-5P in NP cells and Beclin-1 mRNA and protein levels were evaluated by qRT-PCR, western blotting (Figure 2B–2D), and immunofluorescence analyses (Figure 3C, 3D). The results demonstrated that Beclin-1 is negatively regulated by miR-129-5P in the process of autophagy.

### MiR-129-5P regulates NP cell autophagy via regulation of Beclin-1

**Figure 4:**
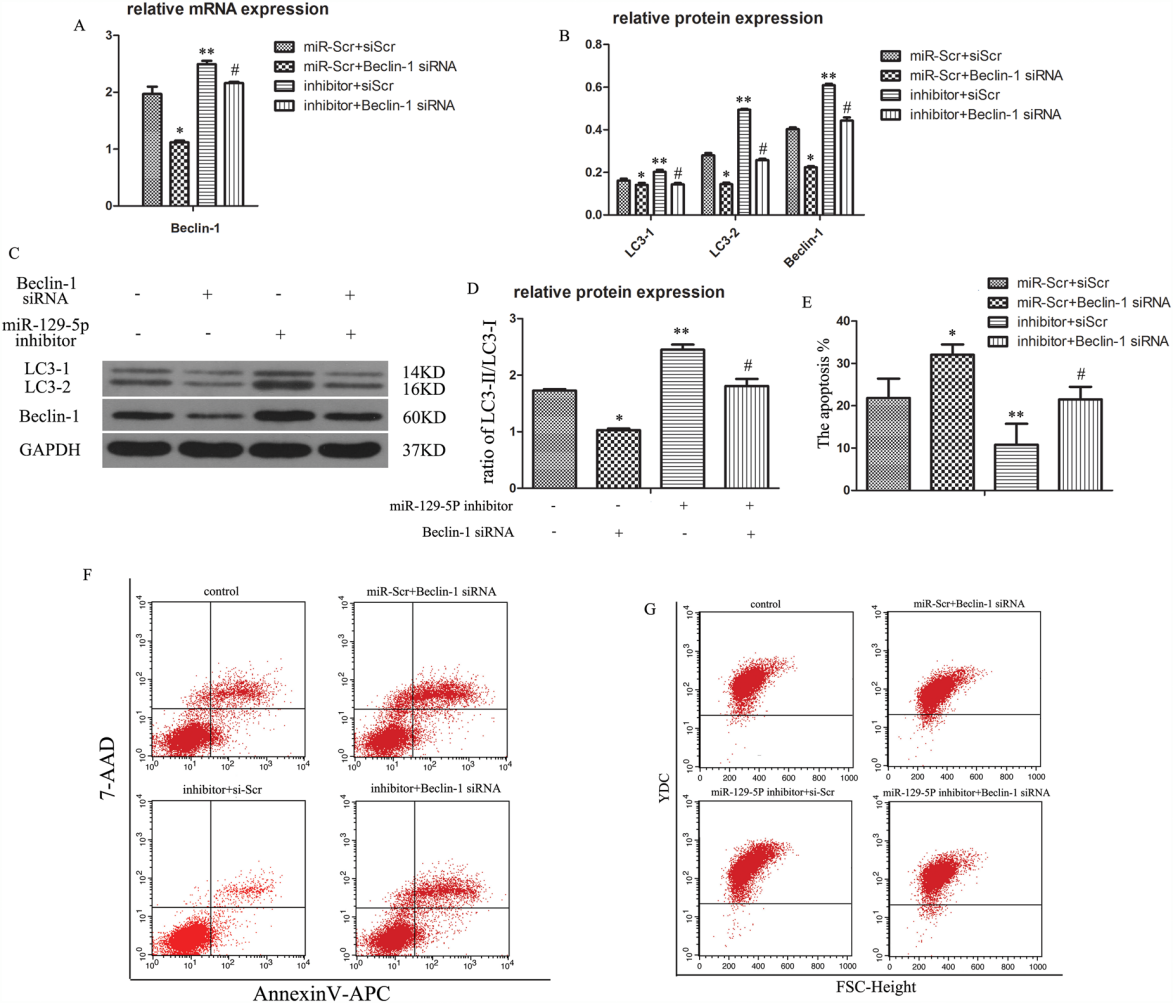
MiR-129-5P regulates degenerative human NP cell autophagy by directly targeting Beclin-1 Human NP cells were transfected with miR-129-5P and Beclin-1 siRNA. **(A)** LC3 and Beclin-1 mRNA expression were measured by qRT-PCR. **(B, C)** The protein of LC3-I, LC3-II, and Beclin-1 in human NP cells, as determined by western blotting. **(D)** LC3-II and LC3-I protein ratio. **(E, F)** Apoptosis of transfected NP cells, as analyzed by flow cytometry. **(G)** Autophagy of treated NP cells, as determined by flow cytometry. Results are shown as mean ± SD. Data are representative of three independent experiments. *P < 0.05 vs. miR-Scr+siScr; **P < 0.05 vs. miR-Scr+siScr; #P < 0.05 vs. inhibitor+siScr.

To verify whether the inhibitory effect of miR-129-5P on NP cell autophagy was exerted via direct targeting of Beclin-1, we inhibited the expression of the two factors by transfection of short interfering (si)RNA constructs (Figure 4A–4C). The ratio of LC3-II to LC3-I was decreased in the Beclin-1 siRNA group (Figure 4D). Meanwhile, Beclin-1 expression increased in the miR-129-5P inhibitor-transfected groups, whereas Beclin-1 siRNA mediated Beclin-1 knockdown reversed this effect (Figure 4A–4C). Similarly, the increase in LC3-II to LC3-I ratio induced by miR-129-5P inhibition was reversed by co-transfection of Beclin-1 siRNA (Figure 4D), which was confirmed by flow cytometry (Figure 4G). These results indicate that Beclin-1 is essential for miR-129-5P-induced autophagy in NP cells. In addition, NP cell apoptosis was increased upon inhibition of autophagy (Figure 4F).

### Autophagy prevents apoptosis by inhibiting cathepsin B release into cytoplasm in human degenerative NP cells

**Figure 5:**
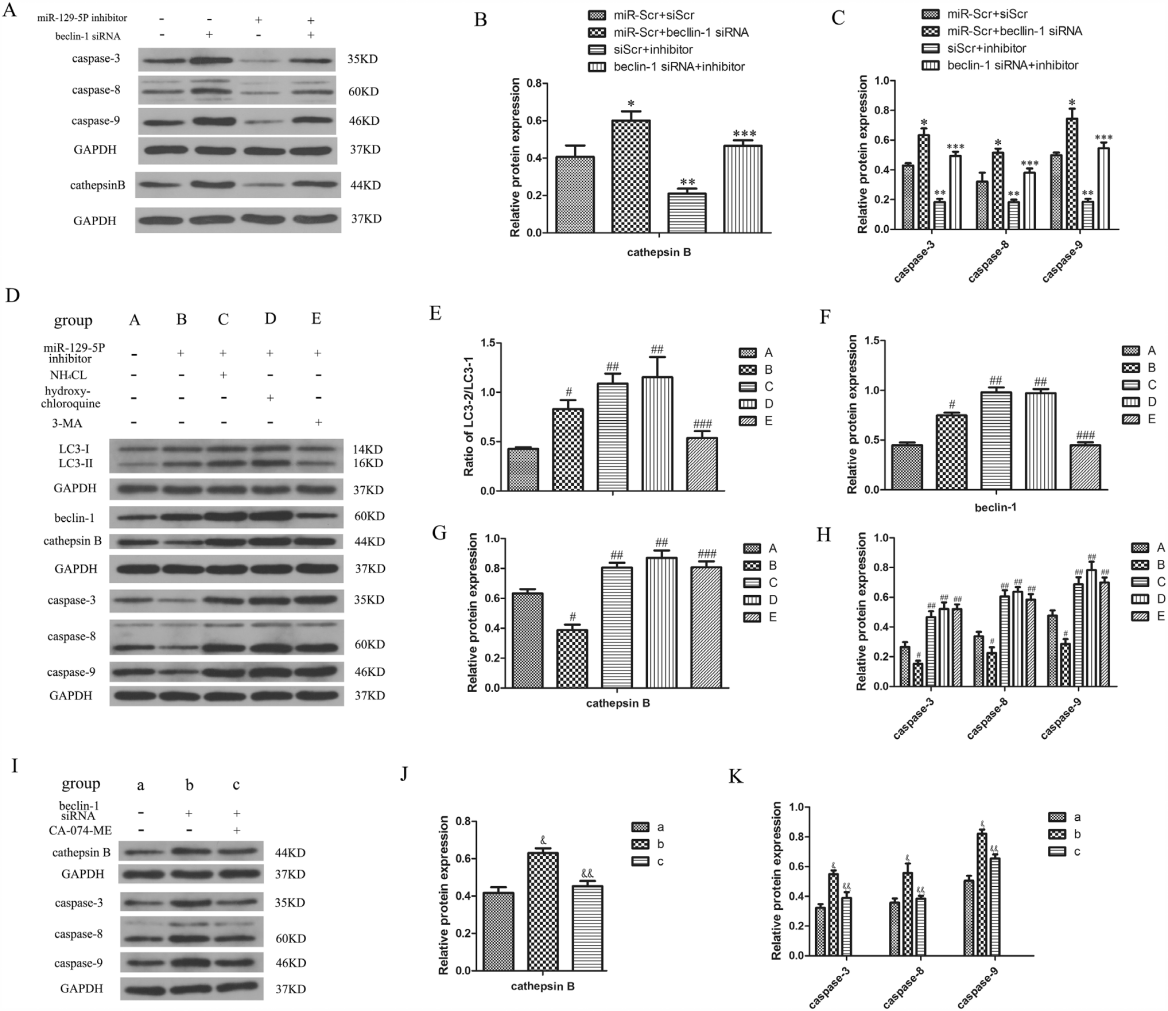
Autophagy inhibits apoptosis by stimulating the cytoplasmic release of cathepsin B in degenerative human NP cells **(A–C)** Caspase-3, -8, -9, and cathepsin B protein expression were determined by western blotting. **(D–H)** Expression of caspases, LC3- II/I, Beclin-1, and cathepsin B protein in NP cells as determined by western blotting following treatment with ammonium chloride, hydroxychloroquine, and 3-MA to block autophagosome and lysosome fusion or autophagosome formation. **(I–K)** Expression of caspases and cathepsin B protein detected by western blotting in cells treated with the cathepsin B inhibitor CA-074-ME. Results are shown as mean ± SD. Data are representative of three independent experiments. *P < 0.05 vs. miR-Scr+siScr; **P < 0.05 vs. miR-Scr+siScr; ***P < 0.05 vs. inhibitor+siScr; #P < 0.05 vs. group A; ##P < 0.05 vs. group B; ###P < 0.05 vs. group B; &P < 0.05 vs. group a; &&P < 0.05 vs. group b.

To investigate the mechanistic basis for the decrease in apoptosis of NP cells when autophagy increased, we evaluated the expression of apoptosis-related proteins in these cells and found that caspase3/8/9 protein expression was increased in the Beclin-1 knockdown group, and decreased in the miR-129-5P inhibitor-transfected group (Figure 5A, 5C). In addition, we found the cathepsin B level in the cytoplasm was increased and decreased in the Beclin-1 knockdown and miR-129-5P inhibitor-transfected groups, respectively (Figure 5A, 5B). These results suggest that cathepsin B level is modulated by NP cell autophagy and is related to their apoptosis. To verify whether cathepsin B is regulated by Beclin-1, we used the autophagy inhibitor ammonium chloride and hydroxychloroquine, which inhibited the fusion of autophysosomes and lysosomes but did not affect Beclin-1 expression, and autophagy inhibitor 3-MA, which inhibited the formation of autophysosomes. We found that apoptosis was increased whereas autophagy was decreased by this treatment, whereas Beclin-1 protein level was increased (Figure 5D–5H). We speculated that Beclin-1 does not directly regulate cathepsin B and that autophagy is direct regulator. To determine whether cathepsin B regulates apoptosis, the cells were treated with CA-074-ME, a cathepsin B-specific inhibitor. Apoptosis was markedly decreased by cathepsin B inhibition (Figure 5I–5K). These results demonstrate that autophagy prevents apoptosis by inhibiting cathepsin B in human degenerative NP cells.

### Methylation inhibits miR-129-5P expression and induces NP cell autophagy

**Figure 6:**
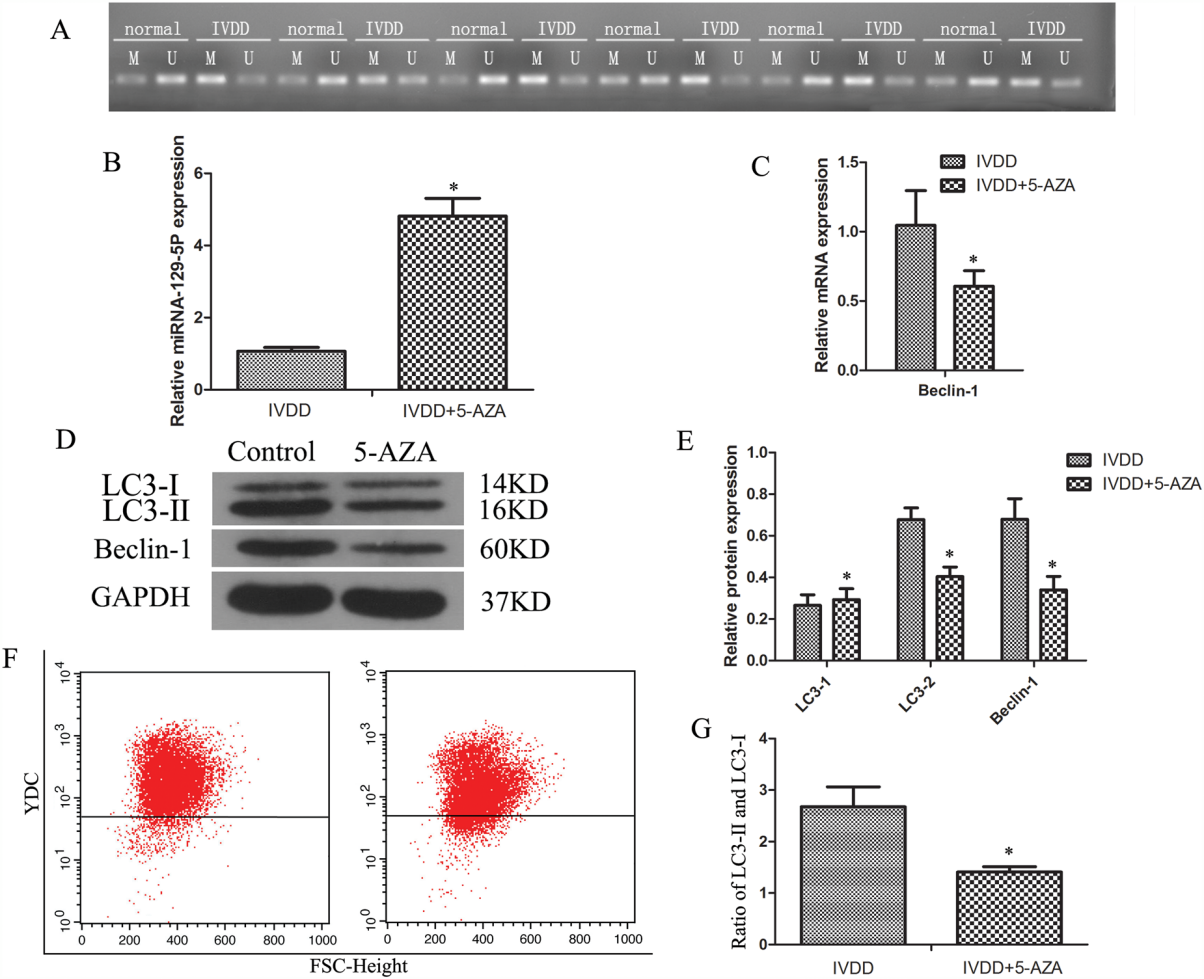
DNA methylation blocked miR-129-5P expression and induced NP cell autophagy in degenerative human NP cells NP cells isolated from IVDD tissue were treated with 5-AZA. **(A)** Methylation status in normal and degenerative NP cells, as detected by MSP. **(B, C)** MiR-129-5P, LC3, and Beclin-1 mRNA levels were measured by qRT-PCR. **(D, E)** LC3-I, LC3-II, and Beclin-1 protein expression in treated NP cells, as determined by western blotting. **(F)** Autophagy in NP cells, as analyzed by flow cytometry. **(G)** LC3-II and LC3-I protein ratio. Results are shown as mean ± SD. Data are representative of three independent experiments. *P < 0.05 vs. IVDD.

The miR-129-5P gene promoter contains CpG islands; we therefore speculated that miR-129-5P expression is regulated by methylation. We verified this possibility in human NP cells with the methylation-specific PCR (MSP) assay and 5-azacitidine (5-AZA) staining. The results of MSP experiment revealed that miR-129-5P promoter methylation was increased in degenerative as compared to normal NP tissue (Figure 6A). To investigate the relationship between miR-129-5P methylation and autophagy, NP cells were treated with the methylation inhibitor 5-AZA. This induced miR-129-5P expression (Figure 6B), while Beclin-1 mRNA levels were also downregulated (Figure 6C). In addition, LC3-II and Beclin-1 protein levels were reduced in 5-AZA treated groups (Figure 6D, 6E), as was the ratio of LC3-II to LC3-I (Figure 6G). A flow cytometry analysis confirmed that autophagy was reduced by 5-AZA treatment (Figure 6F). These results indicate that methylation can inhibit miR-129-5P expression and induce NP cell autophagy in degenerating discs.

## DISCUSSION

It was previously reported that autophagy plays an important role in the pathology of disc degeneration [24, 25]. Autophagy is an evolutionarily conserved mechanism of cellular homeostasis that maintains metabolic balance by clearing damaged proteins and organelles [45] and is thus considered as a pro-survival response to stressors [46]. Autophagy is associated with various diseases [47], and the failure of autophagy causes alterations in articular cartilage that lead to osteoarthritis [45] and disc degeneration [15, 24–26]. Glucosamine was found to induce autophagy via mammalian target of rapamycin signaling as a protective mechanism in NP cells [48] of normal human cartilage [49]. Autophagy has been shown to occur in NP cells [50]; mechanical compression increased the level of Beclin-1 and the conversion of LC3-I to LC3-II [50]. Recent studies have reported that autophagy plays an important role in the progression of disc degeneration [24, 25]. Multiple factors are thought to regulate autophagy, including miRNAs such as miR-129-5P [39, 51–53]. A recent study reported that autophagy was reduced in cartilage tissue and associated with cartilage degeneration [45]. In this study, we found that changes in miR-129-5P and Beclin-1 levels and autophagic activity were associated with disc degeneration. We also observed that autophagy and apoptosis were negatively correlated in human NP cells. This is the first demonstration that miR-129-5P regulates NP cell autophagy by inhibiting Beclin-1 expression.

Increasing evidence suggests that miRNAs regulate diverse biological and pathological processes, including cell growth, differentiation, apoptosis, autophagy, and carcinogenesis. Although miR-129-5P is known to regulate autophagy in atherosclerosis, its expression and role in human NP cell autophagy are unknown.

In this study, we determined that miR-129-5P expression was higher in normal as compared to degenerative NP tissues. We also demonstrated that miR-129-5P inhibited NP cell autophagy. NP cell death involves many changes such as senescence, apoptosis, and autophagy. The last of these has cytostabilizing and anti-apoptotic functions, and plays an important role in IVDD. Autophagy proceeds via Beclin-1-dependent pathway and beclin-1 independent pathway. Beclin-1 contains a Bcl-2-homology (BH)3 domain and has been identified as a BH3-only protein [54]. This protein is essential for autophagy; its coiled-coil domain mediates binding to the class III phosphatidylinositol-3-kinase hVps34. The Beclin-1 complex induces phosphatidylinositol-3-phosphate, which triggers autophagosome formation [55]. Beclin-1 regulates autophagy by mediating Vps34 and other cofactors to form the Beclin-1 complex [56]. MiRNA-129-5P is known to regulate Beclin-1 expression [39], and is thus a potential regulator of autophagy [39, 53]. We speculate that miRNA-119-5p contributes to NP cell apoptosis by targeting Beclin-1.

In this study, we confirmed that miR-129-5P regulates Beclin-1 expression and inhibits autophagy in human NP cells. Transfection of miR-129-5p mimic significantly decreased Beclin-1 expression and inhibited NP cell autophagy, whereas miR-129-5p inhibitor had the opposite effect indicating that miR-129-5p suppresses NP cell autophagy by regulating Beclin-1 expression. Similarly, dysregulated miR-129-5p may prevent autophagy in human IVDD by inhibiting Beclin-1, suggesting that miR-129-5p is an etiological factor in this disease. We also observed that autophagy inhibited apoptosis in human NP cells, although the molecular basis of the relationship between these two processes is unclear.

The fusion of the autophagosome and lysosome is a critical step in autophagy. In addition, the lysosome plays an important role in the progression of apoptosis [57]. Lysosomes contain a variety of proteases, including cysteine cathepsins. In humans, there are 11 cysteine cathepsins including cathepsin B, D, and K that are differentially expressed in various tissues [58]. The release of lysosome cathepsins is linked to a decrease in lysosomal membrane permeabilization [59]. A previous study reported that the release of cathepsin B directly induced apoptosis [34]. We found here that cathepsin B level was positively associated with apoptosis and negatively associated with autophagy in human degenerative NP cells suggesting that cathepsin B is involved in the transition from autophagy to apoptosis. Indeed, administration of autophagy inhibitor that did not affect Beclin-1 expression although cathepsin B was upregulated, suggesting that autophagy regulates cathepsin B release into the cytoplasm. In addition, pharmacological inhibition of cathepsin B significantly reduced NP cell apoptosis. Thus, cathepsin B modulates human NP cell apoptosis and activation of autophagy can prevent NP cell apoptosis by inhibiting cathepsin B release into the cytoplasm.

The regulatory mechanism of miR-129-5P in degenerative discs remains unclear. Increasing evidence demonstrates that epigenetics play an important role in abnormal miRNA expression [42, 43, 60, 61]. DNA methylation of CpG islands in target gene promoters can alter gene expression; for instance, methylation of miR-129-2 has been linked to the regulation of high mobility group box 1 expression in human hepatocellular carcinoma [61]. It is unclear whether miR-129-2 demethylation underlies miR-129-5P overexpression. Three predicted CpG islands are present in the miR-129-2 promoter region (http://www.urogene.org/cgi-bin/methprimer/methprimer.cgi). We speculated that methylation of miR-129-5P promoter CpG islands leads to a decrease in miR-129-5P expression in degenerative NP cells; this was confirmed by MSP analysis of IVDD tissue. We also found that miR-129-5p expression was upregulated by 5-AZA treatment, whereas the levels of the autophagy-related proteins LC3 and Beclin-1 and autophagic activity were decreased. This suggests that miR-129-5P promoter methylation leads to downregulation of miR-129-5P and an increase in autophagic activity in NP cells.

In the current study, we found that miR-129-5P regulated human NP cell autophagy by targeting Beclin-1 and that miR-129-5P expression is modulated by methylation. We also found that autophagy prevented apoptosis in human NP cells. These results provide a basis for IVDD treatment based on overexpression of miR-129-5P.

## MATERIALS AND METHODS

### Patient samples

Human disc samples were collected from 20 patients (10 with degenerative disc disease and 10 with idiopathic scoliosis) (Table 1). Mildly degenerative human discs were obtained from patients with idiopathic scoliosis classified as Pfirrmann grade I or II, as determined by magnetic resonance imaging (MRI) (n = 10; mean age, 24.3 ± 4.25 years; range, 21–40 years). Severely degenerative human disc tissue samples were obtained from patients with lower back pain with disc degeneration classified as Pfirrmann grade IV or V, as determined by MRI (n = 10; mean age, 38.8 ± 5.32 years; range, 25–58 years). The study protocol was approved by the ethics committee of Tongji Medical College, Huazhong University of Science and Technology and conformed to the World Medical Association Declaration of Helsinki Ethical Principles for Medical Research Involving Human Subjects [62].

**Table 1:** Human nucleus pulposus tissue samples

	Pfirrmann grade I/II (n=10)	Pfirrmann grade IV/V (n=10)	P value
**Mean age (y)**	24.3±4.25	38.8±5.32	<0.01
**Sex (male:female)**	3:7	4:6	>0.01
**Diseases**	Idiopathic scoliosis: 10	Lumbar disc herniation: 6 Lumbar spinal stenosis: 4	

### Human degenerative NP cell isolation and culture

Human NP tissue was isolated from mild IVDD tissue under sterile conditions, washed twice with phosphate-buffered saline (PBS), and cut into 1-mm^3^ fragments that were treated with 0.25% trypsin for 30 min and type II collagenase (Invitrogen, Carlsbad, CA, USA) for 3–4 h at 37°C. NP cells were resuspended in Dulbecco’s modified Eagle’s medium (DMEM)/F12 containing 15% fetal bovine serum 100 μg /ml streptomycin, 100 U/ml penicillin, and 1% l-glutamine and incubated at 37°C in a 5% CO_2_ atmosphere. Second-generation of NP cells were used for experiments.

### Cell transfection

MiR-129-5P mimic, scrambled miR-129-5P mimic (control), miR-129-5P inhibitor, scrambled miR-129-5P inhibitor, Beclin-1 siRNA, and scrambled Beclin-1 siRNA were purchased from RiboBio (Guangzhou, China). MiR-129-5P mimic and inhibitor were used to induce and inhibit miR-129-5P expression, respectively, and Beclin-1 siRNA was used to knock down protein expression. Human NP cells were transfected with the constructs using Lipofectamine 2000 (Invitrogen) according to the manufacturer’s instructions and cultured for 48 h before they were used for experiments.

### RNA analysis

Total RNA was extracted from human NP tissue using TRIzol reagent (Aidlab, Beijing, China) according to the manufacturer’s instructions. An ultraviolet spectrophotometer (Shunyu, Shanghai, China) was used to measure RNA purity and concentration. A 7500 real-time PCR instrument (Applied Biosystems, Foster City, CA, USA) was used to quantify miR-129-5P and Beclin-1 and LC3 mRNA levels. The primers used for qRT-PCR are listed in Table 2. U6 was used as the endogenous control. The fold-change in gene expression relative to the control was calculated with the 2^−ΔΔCt^ method.

**Table 2:** Sequences of primers used for RT-PCR and MSP

Gene	Primer	Sequence
MIR129 M	Forward	GTGTGTTTGCGTTTGTTAGTTTC
	Reverse	ACACTATCTTCAAATCCCTATCGAC
MIR129 U	Forward	GTGTGTTTGTGTTTGTTAGTTTTGG
	Reverse	ACACTATCTTCAAATCCCTATCAAC
mir-129-5p	Forward	TGCGCCTTTTTGCGGTCTGGG
	Reverse	CCAGTGCAGGGTCCGAGGTATT
LC3	Forward	GCAGCCTTTGTTCCAGAGAC
	Reverse	CTGGAAAAGTGGAGGCTGAG
Beclin-1	Forward	AGCTGCCGTTATACTGTTCT
	Reverse	TGTGTCTTCAATCTTGCCTT
U6	Forward	CGCTTCGGCAGCACATATAC
	Reverse	AAATATGGAACGCTTCACGA
β-actin	Forward	AGCGAGCATCCCCCAAAGTT
	Reverse	GGGCACGAAGGCTCATCATT

### Western blotting

To analyze protein expression, the culture supernatant was removed, and cells were washed with cold PBS and lysed with radioimmunoprecipitation assay lysis buffer (Beyotime, Beijing, China) for 20 min. The proteins were separated by 10% sodium dodecyl sulfate-polyacrylamide gel electrophoresis and transferred to a polyvinylidene difluoride membrane that was blocked with Tris-buffered saline with 0.1% Tween-20 (TBST) containing 5% non-fat milk for 2 h at room temperature, then incubated overnight at 4°C with antibodies against glyceraldehyde 3-phosphate dehydrogenase (1:1000), Beclin-1 (1:1000), and LC3 (1:1000). After five washes with TBST, the membrane was incubated with a horseradish peroxidase-conjugated secondary goat anti-rabbit IgG antibody for 2 h at 37°C. Immunoreactivity was visualized using an ECL chemiluminescence kit (Thermo Fisher Scientific, Waltham, MA, USA). Protein bands were quantitated using BandScan software (BioMarin Pharmaceutical, San Rafael, CA, USA). The experiment was repeated at least three times.

### Immunofluorescence analysis

NP cells were harvested and washed three times with PBS and fixed with 4% formaldehyde for 15 min at room temperature after transfection with miR-129-5p mimic or inhibitor. Nonspecific binding sites were blocked by incubation with goat serum for 30 min. Cells were incubated overnight at 4°C with anti-Beclin-1 antibody (1:150; Abcam, Cambridge, UK), followed by a 1-h incubation with Cy3-conjugated goat anti-rabbit IgG (GE Healthcare, Little Chalfont, UK; 1:100 dilution) after three washes with PBS at room temperature. Cells were incubated with 4′,6′-diamidino-2-phenylindole (Beyotime, Beijing, China), washed three times, and examined by fluorescence microscopy (Olympus, Tokyo, Japan).

Cells transfected with miR-129-5P mimic or inhibitor were incubated with goat serum for 30 min, followed by overnight incubation at 4°C with primary antibody against LC3 (1:150; Abcam) and a 1-h incubation with secondary antibody (1:100; GE Healthcare). Samples were washed three times with PBS at room temperature, and images were captured on an epifluorescence microscope (Olympus). To quantify autophagy, the percentage of cells with punctuate red fluorescent protein (RFP)-LC3 fluorescence was counted. Cells with more than 10 punctae were considered as undergoing autophagy.

### Bioinformatics analysis and luciferase reporter assay

TargetScan (www.targetscan.org), PicTar (pictar.mdc-berlin.de), and miRanda (www.microrna.org) databases were used to predict potential miR-129-5P targets. Beclin-1 was found to have a putative miR-129-5P binding site. We carried out the luciferase reporter assay to confirm direct binding of miR-129-5P to this site. Beclin-1 3′-UTR reporter plasmids containing the predicted miR-129-5P binding sequence (pRL-Beclin-1 3′-UTR) or a mutation in the miR-129-5p binding site (pRL-Beclin-1 3′-UTR mut) were amplified by PCR and inserted into the pGL3 control vector (RiboBio). NP cells were co-transfected with 200 ng of plasmid along with 90 nM miR-129-5P mimic or scrambled miRNA using Lipofectamine 2000. After culturing for 48 h, NP cells were collected and luciferase activity was detected with the dual-luciferase reporter assay system (Promega, Madison, WI, USA) according to the manufacturer’s instructions. The experiment was repeated at least three times.

### Flow cytometry

Apoptotic NP cells were detected with the Annexin V-allophycocyanin (APC) apoptosis detection kit (BD Pharmingen, San Jose, CA, USA) according to the manufacturer’s instructions. Cultured NP cells were resuspended at a density of 10^5^ cells/ml in PBS and incubated with Annexin V-APC and propidium iodide for 15 min at room temperature in the dark. The cells were analyzed with a fluorescence-activated cell sorter (Beckman Coulter, Miami, FL, USA) over 1 h. Apoptotic cells included those positive for Annexin V-APC and negative for propidium iodide as well as those that were double positive.

NP cell autophagy was evaluated with Acridine Orange (AO) staining. Cells were sub-cultured in 6-well plates at 2 × 10^5^ cells per well. When they reached 90% confluence, the medium was changed to DMEM/F12 containing 1% fetal bovine serum and antibiotics for 12 h in order to synchronize the cells. This was followed by incubation with 1 mM AO in PBS at 37°C for 10 min and four washes with PBS. Intracellular AO was measured by flow cytometry within 30 min.

### MSP assay

Methylation of the miR-129-5P gene promoter was evaluated by MSP analysis in both degenerative and normal NP tissue. Genomic DNA was isolated from NP tissue using a DNA extraction kit (Promega). Sodium bisulfite treatment of DNA was carried out with the Epitect Bisulfite kit (Qiagen, Hilden, Germany) following the manufacturer’s instructions. MSP was carried out using primers that could separately amplify the modified DNA of either the methylated or unmethylated allele. MSP products were separated on a 3% agarose gel containing ethidium bromide and visualized under ultraviolet illumination. Primer sequences used for MSP are shown in Table 2.

### Statistical analysis

Data are expressed as mean ± standard deviation. Statistical analyses were performed using SPSS v.18.0 software (SPSS Inc., Chicago, IL, USA). Mean differences between groups were evaluated with the Student’s t test or analysis of variance. A P value < 0.01 was considered statistically significant.

## Abbreviations

3′-UTR: 3′ untranslated region
5-AZA: 5-azacitidine
AO: Acridine Orange
APC: allophycocyanin
IVDD: intervertebral disc degeneration
LC3: microtubule-associated protein 1 light chain 3
MRI: magnetic resonance imaging
MSP: methylation-specific PCR
NP: nucleus pulposus
PBS: phosphate-buffered saline
qRT-PCR: quantitative real-time PCR
siRNA: short interfering RNA
TBST: Tris-buffered saline with 0.1% Tween-20
